# Reversal of refractory systemic lupus erythematosus-associated immune thrombotic thrombocytopenic purpura and cerebritis via optimized lymphoplasmapheresis: a case report and literature review

**DOI:** 10.3389/fimmu.2026.1841313

**Published:** 2026-07-22

**Authors:** Chi-E Qiu, Yongxia Zhang, Xueyan Xu, Qiongdan Ma, Yonghong Zang, Guosong Jiang

**Affiliations:** 1 Hematology Department, Northeast Yunnan Central Hospital, Zhaotong, Yunnan, China; 2 Department of radiology, Northeast Yunnan Central Hospital, Zhaotong, Yunnan, China; 3 Department of Pulmonary and Critical Care Medicine, Northeast Yunnan Central Hospital, Zhaotong, Yunnan, China; 4 Kunming Medical University, Kunming, Yunnan, China

**Keywords:** ADAMTS13, immune thrombotic thrombocytopenic purpura, lupus cerebritis, lymphoplasmapheresis, systemic lupus erythematosus

## Abstract

**Background:**

Systemic lupus erythematosus (SLE) complicated by immune thrombotic thrombocytopenic purpura (iTTP) and lupus cerebritis is a severe clinical event. Standard therapeutic plasma exchange (TPE) frequently fails in this context due to a “rebound effect”, as it clears circulating antibodies but spares the intravascular reservoir of autoreactive lymphocytes.

**Case presentation:**

A 30-year-old male presented with refractory SLE, severe iTTP (platelet count: 5.00 × 10^9/L; ADAMTS13 activity: 1.49%), and imaging-confirmed lupus cerebritis. The patient underwent a unique “A-B-A” treatment trajectory. While five sessions of standard TPE at an outside facility did not fully halt the hyper-regenerative hemolysis and CNS progression, the implementation of an optimized lymphoplasmapheresis (LPE) regimen at our center induced rapid hematological remission, progressive radiological resolution of brain lesions, and the alleviation of immune-mediated renal injury, culminating in restored renal function and decreased proteinuria.

**Mechanistic insights:**

The optimized LPE protocol utilized a novel dual-parameter modulation (synchronizing blood drawing speed and hematocrit) to precisely extract the pathogenic lymphocyte-rich buffy coat without exacerbating anemia. Paired quantitative analysis of the patient’s peripheral blood and LPE effluent provided direct evidence of substantial cytokine (e.g., IL-10, IL-8, TNF-α) clearance. This facilitated the rapid seroconversion of antinuclear and anti-dsDNA antibodies. Furthermore, this targeted cellular depletion reduced the required allogeneic replacement plasma volume by up to 50% compared with standard TPE, conserving blood bank resources.

**Conclusion:**

Optimized LPE may interrupt the autoimmune cascade by simultaneously reducing circulating humoral mediators and lymphocyte-rich cellular components. This single-case observation supports its potential as a resource-sparing rescue strategy for refractory autoimmune microangiopathy, although further validation is required.

## Introduction

Systemic lupus erythematosus (SLE) complicated by immune thrombotic thrombocytopenic purpura (iTTP) represents a severe, albeit rare (1), clinical overlap driven by severe A disintegrin and metalloproteinase with a thrombospondin type 1 motif, member 13(ADAMTS13) deficiency and intense microvascular thrombosis. When this microangiopathy extends to the central nervous system (CNS)—manifesting as lupus cerebritis—mortality increases significantly (2).

The cornerstone of conventional acute iTTP management involves therapeutic plasma exchange (TPE) and intense immunosuppression (3). However, while TPE effectively clears circulating ADAMTS13 inhibitors and replenishes functional enzymes (3), it primarily addresses downstream humoral products. It does not eliminate upstream cellular drivers: overactive autoreactive B and T cells within the intravascular compartment (4). Consequently, patients with active SLE-driven secondary iTTP frequently experience a refractory clinical course or a “rebound effect,” wherein continuous autoantibody production outpaces standard apheresis clearance (5), culminating in progressive and irreversible organ damage.

Lymphoplasmapheresis (LPE) represents an advanced apheresis modality integrating pathological plasma removal with the targeted physical depletion of pathogenic lymphocytes. By directly reducing the intravascular lymphocyte burden (6), LPE truncates the autoantibody production cycle and mitigates the systemic cytokine storm at its source. Recent comparative cohort studies in severe autoimmune and neuroimmune disorders have suggested that LPE may yield favorable clinical outcomes, greater reductions in pathogenic B cells and inflammatory cytokines, and lower plasma consumption compared with conventional TPE (6, 7).

Herein, we detail an exceptionally rare case of a 30-year-old male presenting with refractory SLE, severe iTTP, and imaging-confirmed lupus cerebritis. Through a unique clinical trajectory encompassing both standard TPE and an optimized LPE protocol, we illustrate the potential value of optimized LPE in achieving hematological remission and radiological improvement of CNS lesions. Furthermore, utilizing paired immunological analyses of peripheral blood and LPE effluent, we provide direct quantitative evidence of LPE’s targeted clearance mechanisms, thereby offering novel therapeutic insights for catastrophic autoimmune microangiopathies.

## Case presentation

A 30-year-old male presented on February 6, 2026, with a 10-day history of jaundice, fatigue, and gingival bleeding, alongside a recent onset of fever. His medical history was notable only for previously cured pulmonary tuberculosis. Physical examination revealed severe mucocutaneous jaundice and scattered petechiae. Initial laboratory investigations demonstrated severe microangiopathic hemolytic anemia (MAHA) and severe thrombocytopenia: hemoglobin (HGB) 64 g/L, reticulocytes (RET) 7.21%, platelets (PLT) 5.00 × 10^9/L, and significantly elevated lactate dehydrogenase (LDH) at 1838 U/L. Concurrent acute kidney injury (creatinine [CRE] 297 μmol/L; blood urea nitrogen [BUN] 20.43 mmol/L) and heavy proteinuria (++++) were evident. A direct antiglobulin test (DAT) was positive, and serum ferritin was markedly elevated at 966 ng/mL.

To address the life-threatening microvascular thrombosis, emergent LPE was initiated on February 6 and 7. This was accompanied by intravenous methylprednisolone (MPO; 40 mg on February 6, escalated to 80 mg on February 7), alkalization, hydration, and the transfusion of 1 unit of suspended red blood cells (RBC) daily. Immunological assays drawn prior to the initial LPE revealed an antinuclear antibody (ANA) titer of 1:160, weakly positive anti-double-stranded DNA (anti-dsDNA), elevated anti-β2 glycoprotein I (163.5 AU/mL), and anti-cardiolipin antibodies >300 AU/mL. Concurrently, the patient exhibited unexplained fever, documented polyserositis (multiple serosal effusions), acute renal involvement (heavy proteinuria), and severe complement consumption (low C3 and C4). These specific clinical and immunological features cumulatively and definitively met the 2019 European League Against Rheumatism/American College of Rheumatology (EULAR/ACR) classification criteria for SLE (8).

Crucially, the patient’s ADAMTS13 activity was severely deficient (1.49%) with detectable circulating inhibitors. According to the International Society on Thrombosis and Haemostasis (ISTH) guidelines, the triad of severe microangiopathic hemolytic anemia, severe thrombocytopenia, and an autoantibody-mediated ADAMTS13 activity of <10%, occurring specifically in the context of an established underlying autoimmune disease, established a definitive diagnosis of secondary immune thrombotic thrombocytopenic purpura (secondary iTTP) (9).

**Figure 1:**
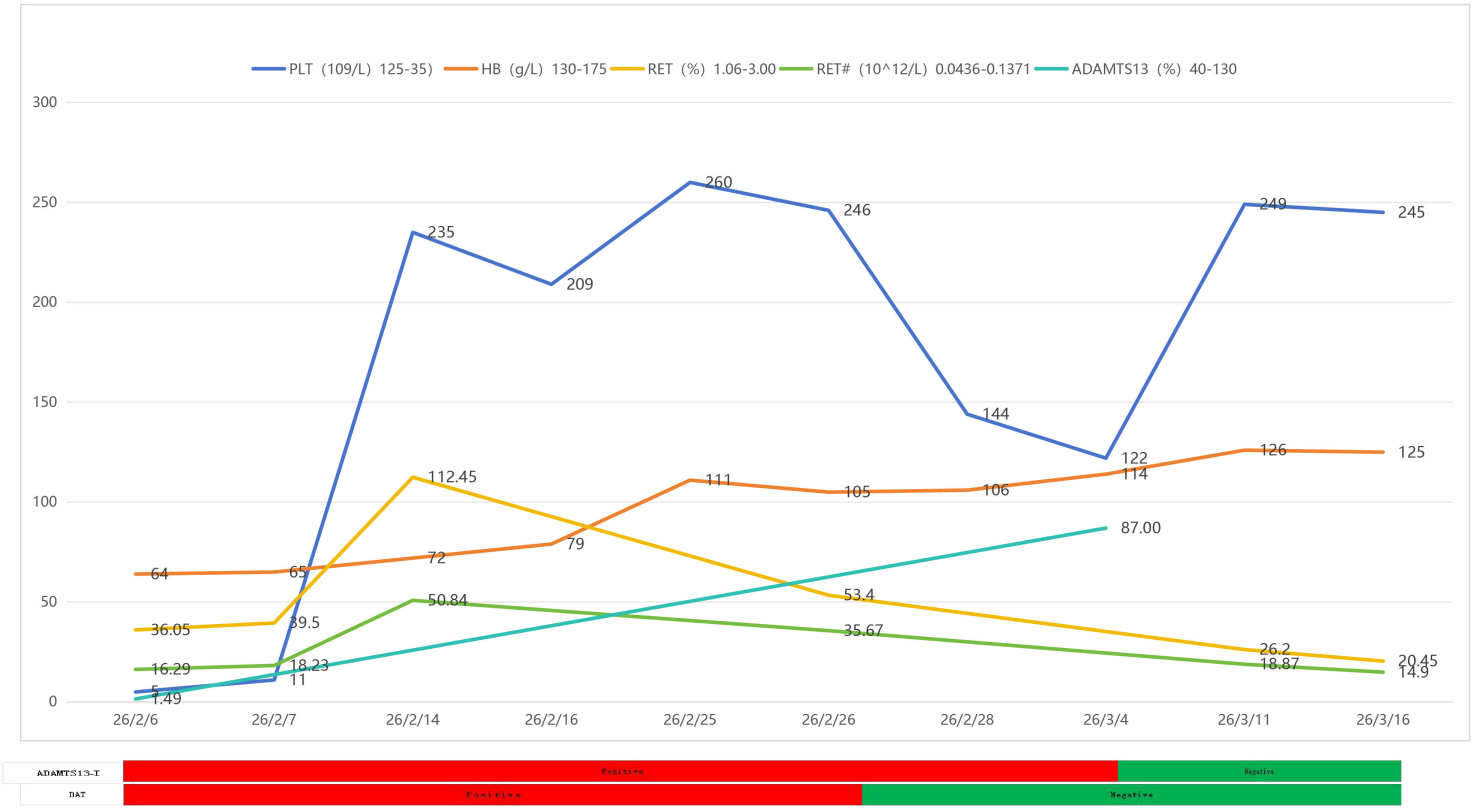
Dynamic changes in hematological and ADAMTS13-related parameters during the treatment course. The line graph shows platelet count, hemoglobin, reticulocyte percentage, absolute reticulocyte count, and ADAMTS13 activity from initial presentation to follow-up. RET% is scaled by a factor of 5, and RET# is scaled by a factor of 100. PLT, platelets; HGB, hemoglobin; RET, reticulocytes; DAT, direct antiglobulin test; TPE, therapeutic plasma exchange; LPE, lymphoplasmapheresis. PLT, ×10^9/L; HGB, g/L; RET%, %; RET#, ×10^12/L; ADAMTS13 activity, %.

At the family’s request, the patient was temporarily transferred to another tertiary hospital from February 8 to 13, where he underwent five sessions of standard TPE. While PLT passively increased to 204 × 10^9/L by February 12, MAHA persisted, evidenced by ongoing anemia (HGB 66.0 g/L) and an elevated reticulocytosis (RET 16.0%) (**Figure 1**).

**Figure 2:**
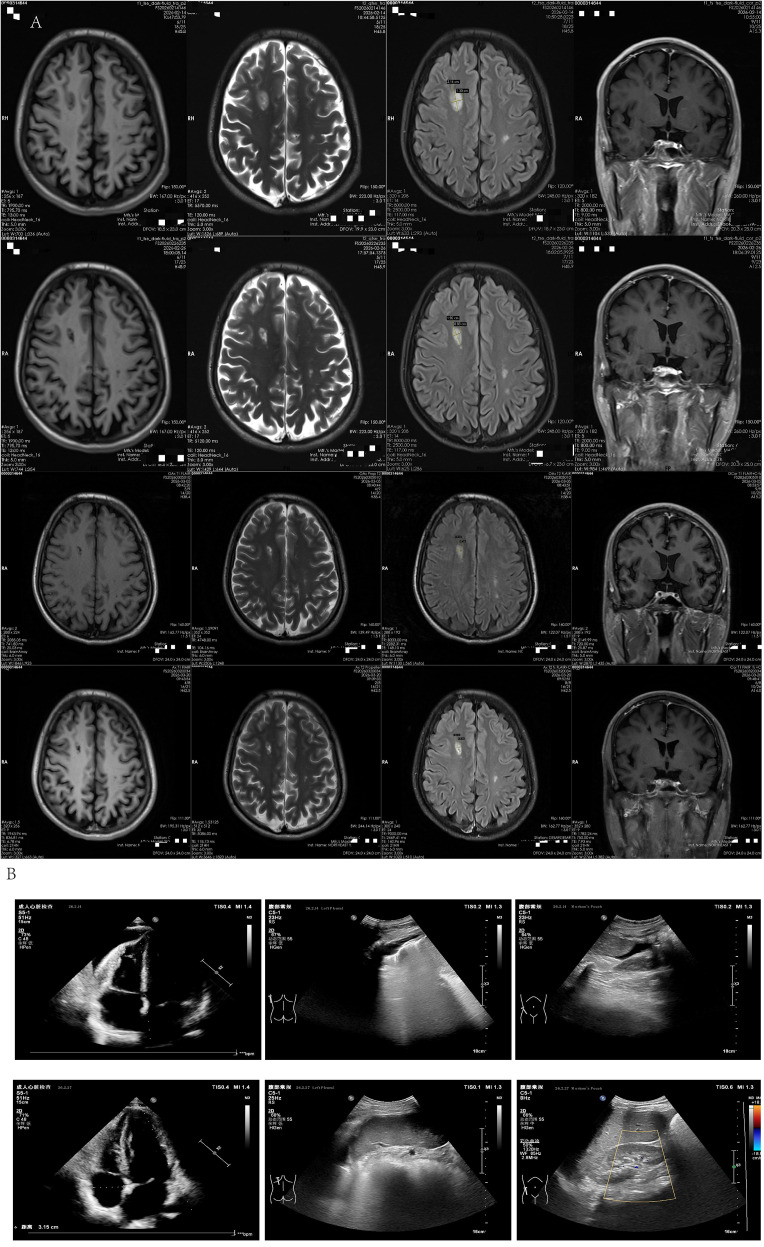
Serial imaging changes in lupus cerebritis and polyserositis. **(A)** Brain MRI images are arranged chronologically from top to bottom: February 14, February 27, March 5, and March 20. Lesion size is reported in centimeters (cm). **(B)** Ultrasonographic images show pericardial, pleural, and abdominal effusions before and after optimized LPE. MRI, magnetic resonance imaging; CNS, central nervous system; LPE, lymphoplasmapheresis.

The patient was readmitted to our center on February 13. Brain magnetic resonance imaging (MRI) on February 14 revealed multiple nodular and patchy abnormal signals in the bilateral periventricular and centrum semiovale regions. The largest lesion (2.1 cm × 1.2 cm) was located in the right centrum semiovale, consistent with lupus cerebritis (**Figure 2A**). Concurrent ultrasonography confirmed multiple serosal effusions (**Figure 2B**).

**Figure 3:**
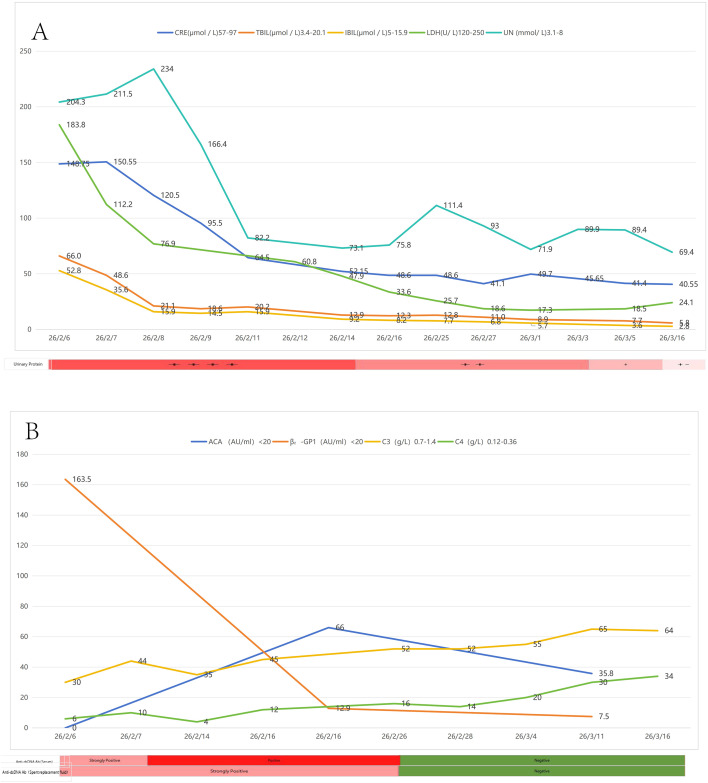
Dynamic recovery of biochemical and systemic immunological parameters. **(A)** Changes in creatinine, urea nitrogen, total bilirubin, indirect bilirubin, lactate dehydrogenase, and semi-quantitative urinary protein. UN is scaled by a factor of 10, CRE by 0.5, and LDH by 0.01. **(B)** Changes in anti-cardiolipin antibodies, anti-β2 glycoprotein I antibodies, C3, C4, and anti-dsDNA status. C3 and C4 are scaled by a factor of 100. CRE, creatinine; UN, urea nitrogen; TBIL, total bilirubin; IBIL, indirect bilirubin; LDH, lactate dehydrogenase; ACA, anti-cardiolipin antibodies; β2-GP1, anti-β2 glycoprotein I; anti-dsDNA, anti-double-stranded DNA; LPE, lymphoplasmapheresis. CRE, μmol/L; UN, mmol/L; TBIL/IBIL, μmol/L; LDH, U/L; ACA and β2-GP1, AU/mL; C3 and C4, g/L; urinary protein, semi-quantitative grade.

To achieve clinical remission and target the pathogenic lymphocyte reservoir, three additional LPE sessions were administered. MPO was reinstituted at 80 mg/day from February 14 to 16 and subsequently tapered. After the reinforced LPE-corticosteroid regimen, platelet counts stabilized within the normal range, hemoglobin gradually recovered, reticulocytosis resolved, ADAMTS13 activity increased to 87.00%, and both ADAMTS13 inhibitors and DAT converted to negative, indicating sustained hematological and immunological remission (**Figure 1**). Biochemical and immunological monitoring showed parallel resolution of hemolysis and immune-mediated organ injury, with normalization of bilirubin and LDH, improvement in creatinine and proteinuria, decline in β2-GP1 levels, rebound of C3/C4, and conversion of anti-dsDNA antibodies to negative in both peripheral blood and LPE effluent (**Figure 3**).

Serial MRI demonstrated progressive absorption of the CNS lesions on February 27, March 5, and March 20, with shrinkage of the largest lesion from 2.1 cm × 1.2 cm to 1.4 cm × 0.7 cm. Follow-up ultrasonography also showed marked absorption of pericardial, pleural, and abdominal effusions, supporting radiological reversal of lupus cerebritis and polyserositis after optimized LPE (**Figure 2**). Following the initial radiological improvement, intravenous belimumab (480 mg) was administered on March 5 to consolidate systemic SLE remission, together with intrathecal dexamethasone (10 mg) and methotrexate (10 mg) on March 5 and March 12. Oral MPO was progressively tapered to 32 mg/day.

A comprehensive, unified chronological record of the patient’s entire clinical progress—detailing all daily laboratory numerical values, respective units, and the exact timing of LPE, TPE, and pharmacological interventions—is provided in **Supplementary Table 1**.

## Materials and methods

### Equipment and anticoagulation protocol

To effectively eradicate circulating autoantibodies and selectively deplete the pathogenic autoreactive lymphocyte reservoir, an optimized lymphoplasmapheresis (LPE) protocol was implemented using the Spectra Optia Apheresis System (Terumo BCT). The procedure was adapted from the standard TPE program. The anticoagulant utilized was Blood Preservative Solution I (comprising 22.0 g sodium citrate, 8.0 g citric acid, and 24.5 g glucose per 1000 mL), administered at a whole blood-to-anticoagulant ratio of 1:12. The replacement fluid across all sessions was strictly O-type Rh-positive virus-inactivated frozen plasma. Comprehensive details regarding clinical laboratory assay methodologies, including inflammatory cytokines, lymphocyte subsets, autoantibodies, and standard biochemical parameters, are provided in **Supplementary Text S1**.

During the treatment course, nine LPE sessions were performed. To achieve strict hemodynamic stability, a precise net-negative fluid balance was maintained: the actual removed effluent volume per session exceeded the infused replacement plasma volume by precisely 50 to 100 mL. This intentional differential directly accounted for the volume of anticoagulant entering the patient’s systemic circulation, ensuring meticulous euvolemia.

### Optimized dual-parameter modulation technique

Conventional centrifugation methods for lymphocyte separation (such as standard mononuclear cell apheresis) are primarily designed for routine WBC collection. They typically return the patient’s native plasma—leaving circulating autoantibodies intact—and rely strictly on automated optical sensors to maintain the cell separation interface. However, in patients presenting with severe microangiopathic hemolytic anemia, these conventional automated systems frequently fail to maintain a stable interface, resulting in either inadequate buffy coat extraction or substantial iatrogenic RBC loss.

Our optimized LPE protocol overcomes these limitations by differing fundamentally in both therapeutic scope and technical execution. The entire optimized extraction procedure was executed through a precise 10-step protocol (**Supplementary Figure 1**).

First, unlike conventional methods that return native plasma, LPE achieves simultaneous plasma exchange and cellular depletion. After standard system power-on, the operator specifically selects the Plasma Exchange (PE) program (**Supplementary Figure 1**, Step 1) and utilizes the 10226 tubing set (Step 2).

Second, the target processed whole blood volume was preset to 10,000–15,000 mL, exceeding the planned exchange volume for the procedure (Step 3). This setting helped maintain procedural continuity and reduce repeated interruption of the separation interface by automated return cycles.

In patients with severe microangiopathic hemolytic anemia, automated optical interface detection may become unstable because of abnormal blood-component characteristics. Therefore, the Spectra Optia TPE program was operated in the manufacturer-described semi-automatic mode, allowing operator-controlled interface management using entered hematocrit and other procedural values. Interface-related alarms prompted immediate operator assessment, visual confirmation of the separation interface and effluent line, verification of patient and circuit status, and continuation only when interface stability and patient/circuit safety could be assured. Detailed manufacturer-manual excerpts, safety monitoring, and termination criteria are provided in **Supplementary Text S2**.

After interface stabilization, two-stage operator-controlled dual-parameter modulation was performed. First, the HCT was fine-tuned to maintain the plasma extraction line color between levels 1 and 3 (Step 7). Subsequently, the blood collection speed (inlet flow rate) was delicately adjusted to strictly narrow and maintain the plasma line color between levels 1 and 2 (Step 8). Upon achieving the target exchange volume, reinfusion was initiated, followed by safe disconnection (Steps 9–10).

By synergistically modulating these two variables, the operator stabilized the extraction line color within the target colorimetric range of levels 1 to 2. This visual threshold, manifested as a stable pale-red cellular effluent, was used as a real-time indicator of lymphocyte-rich effluent extraction while minimizing sustained RBC contamination in a setting where automated interface control was unstable.

### Mechanistic findings and discussion

The confluence of active SLE and iTTP represents a severe immunologic crisis. This dual pathology is driven by a severe breakdown in immune tolerance, leading to the significant production of ADAMTS13 inhibitors and subsequent widespread microvascular thrombosis (10–12). When this microangiopathy compromises the blood-brain barrier—manifesting as lupus cerebritis—the mortality rate is high (13).

A critical diagnostic challenge herein was the initial presentation of severe MAHA alongside a positive DAT. In patients with SLE, a positive DAT frequently misleads clinicians toward diagnosing autoimmune hemolytic anemia (AIHA) or Evans syndrome, potentially delaying life-saving interventions (14). This case underscores the clinical imperative that in severe SLE complicated by severe thrombocytopenia and MAHA, prompt screening for ADAMTS13 activity and its inhibitors is mandatory, regardless of DAT positivity.

The unique “A-B-A” (LPE-TPE-LPE) clinical trajectory of this patient provides an observational illustration of the potential mechanistic advantage of LPE over standard TPE. While standard TPE effectively removes circulating antibodies and replenishes functional ADAMTS13, it spares the intravascular reservoir of pathogenic lymphocytes. Consequently, autoantibodies and inflammatory mediators rapidly redistribute and regenerate—a phenomenon known as the “rebound effect”. This was clinically evident during the patient’s transfer to an outside facility, where five sessions of TPE failed to arrest the hyper-regenerative hemolysis or prevent the progression of CNS lesions.

The insufficiency of TPE in addressing the cellular compartment was suggested by immunological profiling at readmission. After five sessions of TPE, total lymphocyte and B-cell counts were 877.89 cells/μL and 339.92 cells/μL, respectively (**Supplementary Figure 2**). We do not interpret these absolute cell counts as established standalone indicators of SLE disease activity. Rather, they were considered exploratory cellular kinetic data reflecting the intravascular lymphocyte compartment potentially targeted by optimized LPE. The inference of ongoing active SLE was mainly supported by conventional clinical and serological findings, including persistent complement consumption, strongly positive anti-dsDNA antibodies, renal and hematological involvement, and progressive CNS lesions on MRI. After optimized LPE, the decline in lymphocyte and B-cell counts paralleled serological, hematological, and radiological improvement, supporting a cellular-depletion effect rather than defining SLE activity by cell counts alone.

By contrast, LPE bridges this therapeutic and logistical gap by integrating plasma exchange with the targeted physical depletion of the lymphocyte-rich buffy coat, acting directly at the source of antibody production. This mechanistic advantage is supported by recent comparative cohort studies. For instance, Duan et al. demonstrated that in patients with myasthenic crisis, LPE significantly reduced the duration of mechanical ventilation and intensive care unit stay compared to TPE, exhibiting superior performance in decreasing autoantibodies and inflammatory cytokines (7). Similarly, a multicenter study by Zeng et al. in neuromyelitis optica spectrum disorder (NMOSD) confirmed that LPE significantly reduced the proportions of memory B cells and plasmablasts, lowered pro-inflammatory cytokines, and achieved substantial clinical improvement (6).

Beyond its potential cellular-depletion effect, our LPE protocol may offer a resource-sparing advantage by reducing the required volume of donor replacement plasma by one-third to one-half (33%–50%) compared with standard TPE requirements. Given the global scarcity of blood products, this substantial reduction not only conserves invaluable blood bank resources but also minimizes the patient’s exposure to allogeneic plasma, thereby mitigating the inherent risks of volume overload, allergic reactions, and transfusion-related acute lung injury (15).As evidenced herein, optimizing the LPE protocol via dual-parameter modulation translates this theoretical advantage—maximizing targeted immunological clearance while minimizing allogeneic blood consumption—into clinical efficacy, successfully reversing refractory hematological consumption where conventional TPE encountered a therapeutic plateau.

Optimized LPE remains an operator-dependent rescue strategy rather than a routine replacement for standard TPE. To avoid red blood cell contamination, iatrogenic blood loss, unstable separation, citrate-related complications, or delayed recognition of circuit problems, this procedure should be restricted to experienced apheresis teams under direct physician supervision, with predefined criteria for termination or conversion to standard TPE.

**Figure 4:**
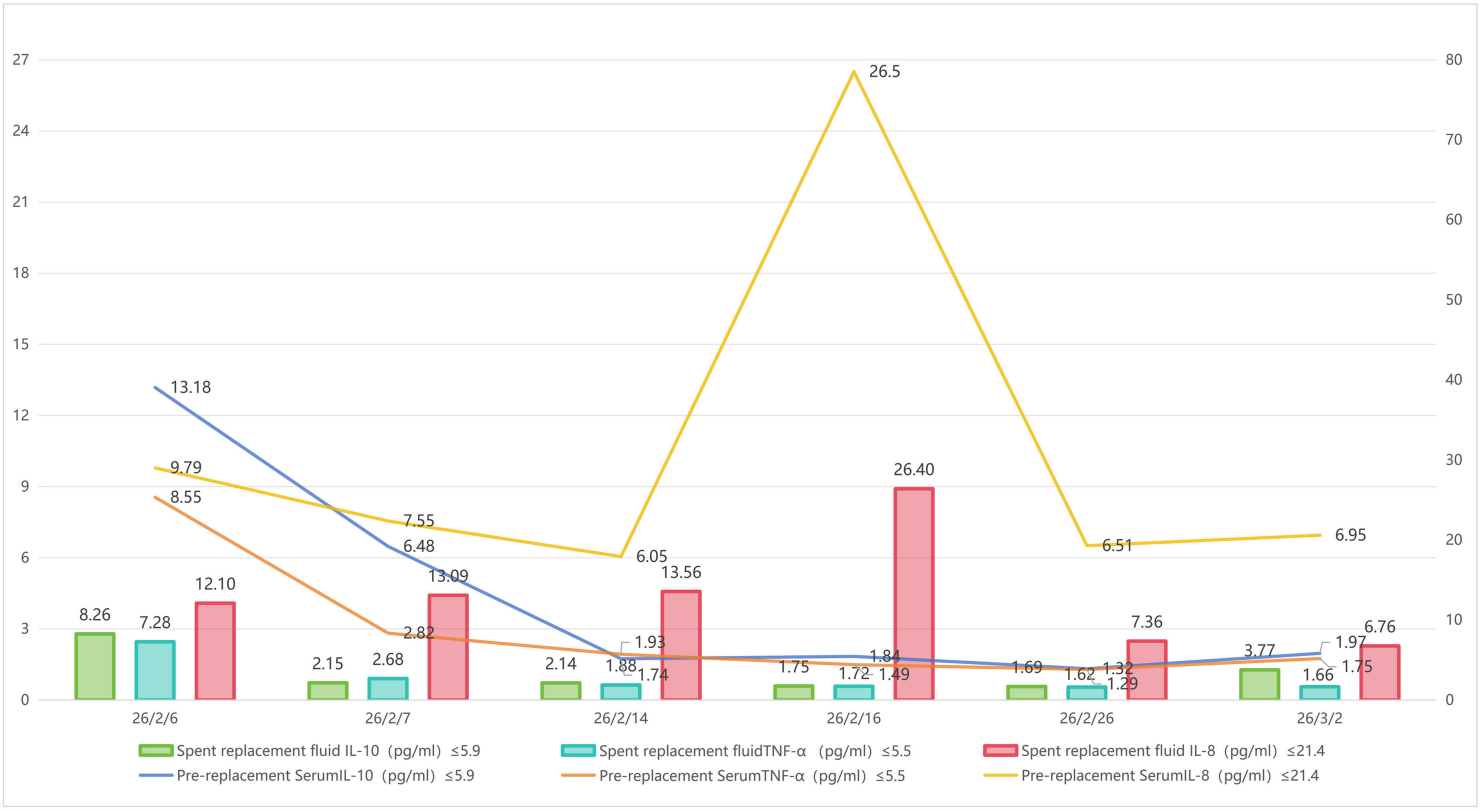
Paired analysis of inflammatory cytokines in peripheral serum and LPE effluent. Lines indicate pre-replacement peripheral serum concentrations of IL-10, TNF-α, and IL-8; bars indicate corresponding cytokine concentrations in the LPE effluent. Cytokine concentrations are reported in pg/mL, and reference ranges are shown in the figure legend. IL-10, interleukin-10; TNF-α, tumor necrosis factor-alpha; IL-8, interleukin-8; LPE, lymphoplasmapheresis.

To further explore the clearance mechanism, we performed paired immunological analysis of peripheral serum and LPE effluent. IL-10, IL-8, and TNF-α were detectable in the LPE effluent, while their peripheral serum concentrations generally declined during treatment, supporting the physical removal of circulating inflammatory mediators by optimized LPE (**Figure 4**). Qualitative immunological analysis also detected high-titer ANA and anti-dsDNA antibodies in the LPE effluent. After the reinforced LPE regimen, peripheral ANA and anti-dsDNA antibodies converted to negative, providing supportive evidence that optimized LPE contributed to both inflammatory mediator removal and autoantibody clearance. Together, these findings support the interpretation that optimized LPE may contribute to both humoral mediator removal and lymphocyte-rich cellular depletion, thereby facilitating serological improvement and radiological resolution of lupus cerebritis.

## Literature review

The conventional cornerstone for managing acute iTTP relies heavily on TPE combined with intense pharmacological immunosuppression (16). A single standard TPE session typically clears approximately 63% of the target pathogenic substances from the circulating blood (17). While TPE is highly effective at immediately removing circulating ADAMTS13 inhibitors, its mechanistic scope is strictly limited to the extracellular humoral compartment, precipitating the well-documented “rebound effect” (18). More importantly, standard TPE fails to address the upstream cellular drivers; the intravascular reservoir of hyperactive autoreactive lymphocytes remains entirely intact.

Furthermore, conventional TPE is highly resource-intensive, typically requiring the exchange of 1.0 to 1.5 plasma volumes per session. This demand places a substantial strain on blood bank resources, particularly during critical shortages (19). While targeted biological therapies (e.g., rituximab) effectively deplete B cells, their delayed onset of action (typically 1 to 3 weeks) renders them suboptimal as standalone rescue therapies in fulminant crises (20).

LPE may help bridge this therapeutic and logistical gap as a mechanistically complementary apheresis modality. Building upon the foundation of conventional plasma exchange, LPE incorporates a dedicated lymphocyte separation and removal step. By physically extracting lymphocytes (21), LPE further depletes the autoantibody-producing B lymphocytes and the T lymphocytes that regulate the pathological immune response. Unlike TPE, which merely clears pre-formed circulating autoantibodies, LPE acts directly at the source of antibody production (22).

Crucially, by strategically concentrating and extracting the pathogenic targets within the buffy coat, LPE achieves superior immunological clearance while simultaneously decreasing the required volume of replacement plasma by up to 50%. This dual benefit—maximizing immune modulation while minimizing allogeneic blood consumption—provides a clinical advantage in managing severe autoimmune diseases. As observed in this case, optimization of the LPE protocol via dual-parameter modulation may translate the theoretical advantages of LPE into clinical benefit, contributing to the improvement of refractory hematological consumption and severe CNS lesions after an inadequate response to conventional TPE. Future large-scale randomized controlled trials are warranted to further delineate the exact efficacy and health-economic profile of LPE across diverse autoimmune microangiopathies.

## Conclusion

This case suggests that optimized lymphoplasmapheresis may provide a rescue strategy for refractory SLE-associated iTTP complicated by lupus cerebritis by simultaneously removing circulating humoral mediators and lymphocyte-rich cellular components. The clinical, radiological, hematological, and immunological improvements observed after reinforced LPE support its potential value in severe autoimmune microangiopathy when standard TPE is insufficient. However, this observation is based on a single case, and optimized LPE should be applied only by experienced apheresis teams under strict safety protocols. Further multicenter studies are needed to define its efficacy, safety, and optimal indications.

## Data Availability

The original contributions presented in the study are included in the article/**Supplementary Material**. Further inquiries can be directed to the corresponding author.

## Glossary

ADAMTS13: A disintegrin and metalloproteinase with a thrombospondin type 1 motif, member 13
AIHA: Autoimmune hemolytic anemia
AIM: Automated Interface Management
ANA: Antinuclear antibody
anti-dsDNA: Anti-double-stranded DNA
BUN: Blood urea nitrogen
CNS: Central nervous system
CRE: Creatinine
DAT: Direct antiglobulin test
HCT: Hematocrit
HGB: Hemoglobin
IL-8: Interleukin-8
IL-10: Interleukin-10
iTTP: Immune thrombotic thrombocytopenic purpura
LDH: Lactate dehydrogenase
LPE: Lymphoplasmapheresis
MAHA: Microangiopathic hemolytic anemia
MPO: Methylprednisolone
MRI: Magnetic resonance imaging
PLT: Platelets
RBC: Red blood cell
RET: Reticulocytes
SLE: Systemic lupus erythematosus
TNF-α: Tumor necrosis factor-alpha
TPE: Therapeutic plasma exchange.
