# Supplementary material for: Reversal of refractory systemic lupus erythematosus-associated immune thrombotic thrombocytopenic purpura and cerebritis via optimized lymphoplasmapheresis: a case report and literature review
Source: Front Immunol. 2026 Jul 22;17:1841313. doi: 10.3389/fimmu.2026.1841313 (PMC13437795; doi:10.3389/fimmu.2026.1841313)
Supplement: Supplementary file 1 [file Table1.docx]

**Supplementary Figure S1. Standardized procedural flowchart and safety checkpoints for the optimized lymphoplasmapheresis (LPE) protocol.**

This flowchart summarizes the stepwise implementation of optimized LPE using the Spectra Optia therapeutic plasma exchange program, including system power-on, tubing installation, parameter entry, operation initiation, alarm assessment, hematocrit (HCT) readjustment, HCT fine-tuning, blood collection speed adjustment, target exchange volume realization, and system power-off. The protocol applies operator-controlled semi-automatic interface management to maintain a stable lymphocyte-rich effluent while minimizing red blood cell (RBC) contamination. Interface-related alarms require immediate assessment of the alarm type, visual inspection of the separation interface and effluent line, verification of patient and circuit status, and continuation only when the interface remains stable and patient safety can be assured. Persistent RBC contamination, recurrent unexplained alarms, or failure to stabilize the interface should prompt immediate termination of optimized LPE or conversion to standard therapeutic plasma exchange (TPE). This procedure should be performed only by experienced therapeutic apheresis personnel under direct physician supervision. LPE, lymphoplasmapheresis; TPE, therapeutic plasma exchange; HCT, hematocrit; RBC, red blood cell.
