# Supplementary material for: Reversal of refractory systemic lupus erythematosus-associated immune thrombotic thrombocytopenic purpura and cerebritis via optimized lymphoplasmapheresis: a case report and literature review
Source: Front Immunol. 2026 Jul 22;17:1841313. doi: 10.3389/fimmu.2026.1841313 (PMC13437795; doi:10.3389/fimmu.2026.1841313)
Supplement: Supplementary file 2 [file Table2.docx]

Figure S2. Dynamic changes in peripheral blood lymphocyte subsets during treatment.

The line graph shows absolute counts of total lymphocytes, T cells, B cells, CD4+ T cells, CD8+ T cells, natural killer cells, and the CD4+/CD8+ ratio. Total lymphocyte and B-cell counts were relatively high at readmission after standard TPE and declined after optimized LPE. These changes are presented as exploratory cellular kinetic observations reflecting the lymphocyte compartment targeted by LPE, rather than as standalone indicators of SLE disease activity. Cell counts are reported as cells/μL; the CD4+/CD8+ ratio is dimensionless. TPE, therapeutic plasma exchange; LPE, lymphoplasmapheresis; NK, natural killer.
