# Supplementary material for: Reversal of refractory systemic lupus erythematosus-associated immune thrombotic thrombocytopenic purpura and cerebritis via optimized lymphoplasmapheresis: a case report and literature review
Source: Front Immunol. 2026 Jul 22;17:1841313. doi: 10.3389/fimmu.2026.1841313 (PMC13437795; doi:10.3389/fimmu.2026.1841313)
Supplement: Supplementary file 3 [file Table4.docx]

**Supplementary Text S1: Detailed Laboratory Assay Methodologies**

**1. Immunological and Inflammatory Marker Assays**

- **12-Panel Inflammatory Cytokines (including IL-10, IL-8, TNF-α):** Analyzed using multiplex cytometric bead array (CBA) flow cytometry.
  - *Reagent:* Boshengte 12-plex Cytokine Assay Kit.
  - *Equipment:* Zhonghan Shengtai iMatrix100 Flow Cytometry Multiplex System.
- **T Lymphocyte Subsets:** Analyzed via flow cytometry.
  - *Reagent:* RaiseCare Diagnostic Reagents.
  - *Equipment:* RaiseCyte 2L6C Flow Cytometer.
- **Antinuclear Antibodies (ANA):** Detected using indirect immunofluorescence (IIF).
  - *Reagent:* EUROIMMUN ANA IgG Assay Kit.
  - *Equipment:* Manual qualitative evaluation.
- **ANA Profile (15 Items):** Detected using immunoblotting assay.
  - *Reagent:* YHLO ANA Profile Assay Kit.
  - *Equipment:* YHLO Tenfly Phoenix system.
- **Five Routine Immune Items (Immunoglobulins & Complements):** Analyzed via immunoturbidimetry (transmission/scattering).
  - *Reagent:* Guangzhou Kefang Biotechnology Co., Ltd.
  - *Equipment:* Fully Automated Biochemical Analyzer.
- **Direct Antiglobulin Test (DAT):** Performed using microcolumn gel technology.
  - *Reagent:* Anti-human globulin (IgG+C3d) test card.
  - *Equipment:* Specialized reagent card incubator and medical centrifuge.

**2. Standard Hematological Assays**

- **Hemoglobin (HGB), Red Blood Cells (RBC), and Platelets (PLT):** HGB was measured using colorimetry (Lambert-Beer law). RBC and PLT counts were determined via the sheath flow impedance method. Mean Corpuscular Volume (MCV) and Red Cell Distribution Width (RDW) were derived indirectly from the RBC histogram.
  - *Reagent:* Mindray Diagnostics.
  - *Equipment:* Mindray BC-6800Plus Automated Hematology Analyzer.

**3. Biochemical and Endocrinological Assays**

- **Total and Indirect Bilirubin (TBIL/IBIL):** Measured using the vanadate oxidation method.
- **Lactate Dehydrogenase (LDH) and Urea Nitrogen (BUN):** Measured using the kinetic rate method.
- **Serum Creatinine (CRE):** Measured using the enzymatic sarcosine oxidase method.
  - *Reagents & Equipment for the above:* Guangzhou Kefang Biotechnology Co., Ltd. / Fully Automated Biochemical Analyzer.
- **Qualitative Urine Protein:** Analyzed via urine dry chemistry.
  - *Reagent:* 9A Urine Test Strips.
  - *Equipment:* UC-3500 Automated Urine Chemistry Analyzer.
- **Thyroid Function:** Analyzed via direct chemiluminescence immunoassay.
  - *Reagent:* Siemens Atellica IM.
  - *Equipment:* Siemens Atellica IM 1600 Analyzer.
- **25-Hydroxyvitamin D [25(OH)D]:** Analyzed via chemiluminescence immunoassay.
  - *Reagent:* Maccura Biotechnology.
  - *Equipment:* Maccura i3000 Automated CLIA Analyzer.

**4. Optimized Lymphoplasmapheresis (LPE) Procedural Note**

- **Spectra Optia Apheresis System:** The procedure utilized the Therapeutic Plasma Exchange (TPE) program. By synergistically adjusting operational parameters, the connector interface was specifically elevated and maintained at a targeted height (as visually demonstrated in **Supplementary Figure S1**). This allowed the concurrent physical depletion of pathogenic lymphocytes alongside plasma exchange, thereby executing the optimized LPE therapeutic effect.
