# Supplementary material for: Reversal of refractory systemic lupus erythematosus-associated immune thrombotic thrombocytopenic purpura and cerebritis via optimized lymphoplasmapheresis: a case report and literature review
Source: Front Immunol. 2026 Jul 22;17:1841313. doi: 10.3389/fimmu.2026.1841313 (PMC13437795; doi:10.3389/fimmu.2026.1841313)
Supplement: Supplementary file 4 [file Table5.docx]

**Supplementary Text S2. Manufacturer-documented basis and safety safeguards for semi-automatic interface management during optimized lymphoplasmapheresis**

**1. Manufacturer-documented technical background**

The optimized lymphoplasmapheresis (LPE) protocol was performed using the Spectra Optia therapeutic plasma exchange program. The Spectra Optia Operator’s Manual describes the system as an automatic blood component separator that uses centrifugation and optical detection to perform therapeutic apheresis and cell collection procedures. The touch-screen interface allows the operator to enter patient and procedure data, perform the procedure, troubleshoot conditions, and adjust run values to achieve a specific procedural outcome.

The same manual describes the Automated Interface Management (AIM) system as an optical detection system that monitors and interprets the separation interface within the connector. AIM has three main functions: monitoring the interface position, interpreting the interface position by measuring transmitted light intensity, and adjusting plasma pump flow to maintain the optimal interface position.

The operator’s manual also describes semi-automatic mode. In this mode, AIM does not control the interface position; instead, the system controls the interface using the entered hematocrit and other procedural values. The manual further states that semi-automatic mode may be entered under certain procedure conditions, and that under selected alarm conditions the operator may be required to disable AIM and complete the procedure in semi-automatic mode. Therefore, the key technical step in optimized LPE should be understood as operator-controlled semi-automatic interface management within a manufacturer-described workflow, not as unsupervised overriding of device safety functions.

**2. Institutional implementation of optimized LPE**

In this case, semi-automatic interface management was used because severe microangiopathic hemolytic anemia may interfere with automated optical interface detection and cause unstable separation. After interface-related alarm assessment, the operator visually inspected the connector and effluent line, verified patient and circuit status, and continued only when the separation interface was stable. Hematocrit setting and inlet blood flow rate were then adjusted to maintain a pale-red lymphocyte-rich effluent while avoiding sustained red blood cell contamination.

**3. Operator requirements and monitoring**

Optimized LPE was performed only by experienced therapeutic apheresis personnel under direct physician supervision. Continuous monitoring included vital signs, vascular access status, circuit pressure and flow parameters, effluent color, fluid balance, symptoms of citrate toxicity, and visual stability of the separation interface. Laboratory surveillance included hemoglobin, hematocrit, platelet count, electrolytes, calcium status when clinically indicated, coagulation profile, and biochemical markers of hemolysis and organ injury.

**4. Potential hazards and safety-stop criteria**

Potential hazards include red blood cell contamination, iatrogenic blood loss, worsening anemia, unstable cell separation, hemodynamic instability, citrate-related hypocalcemia, allergic or transfusion reactions, vascular access complications, bleeding, infection, and procedure failure. Predefined safety-stop criteria included persistent red blood cell contamination, recurrent unexplained alarms, inability to maintain a stable interface, symptomatic hypotension, severe citrate reaction or hypocalcemia, allergic or transfusion reaction, vascular access dysfunction, or any operator concern regarding patient or circuit safety. Under these circumstances, optimized LPE should be immediately terminated or converted to standard therapeutic plasma exchange.

**5. Safety observation in the present case**

All nine LPE sessions in the present case were completed under continuous monitoring. No procedure-related serious adverse event, including sustained hemodynamic instability, clinically significant hypocalcemia, allergic reaction, vascular access complication, persistent red blood cell contamination, or abrupt hemoglobin decline attributable to the procedure, was observed.
